# Self-Assembly Synthesis of the MoS_2_/PtCo Alloy Counter Electrodes for High-Efficiency and Stable Low-Cost Dye-Sensitized Solar Cells

**DOI:** 10.3390/nano10091725

**Published:** 2020-08-31

**Authors:** Zhi Zeng, Dongbo Wang, Jinzhong Wang, Shujie Jiao, Yuewu Huang, Sixiang Zhao, Bingke Zhang, Mengyu Ma, Shiyong Gao, Xingguo Feng, Liancheng Zhao

**Affiliations:** 1 Department of Optoelectronic Information Science, School of Materials Science and Engineering, Harbin Institute of Technology, Harbin 150001, China; zengzhi_1997@163.com (Z.Z.); huangyuewu703@163.com (Y.H.); auspice123@163.com (S.Z.); zhangbingke007@163.com (B.Z.); wangdong165@sina.com (M.M.); gaoshiyong@hit.edu.cn (S.G.); lczhao@hit.edu.cn (L.Z.); 2 National Key Laboratory of Science and Technology on Surface Engineering, Lanzhou Institute of Physics, Lanzhou 730000, China

**Keywords:** MoS_2_/PtCo-alloy nanoparticles, dye-sensitized solar cells, counter electrodes, efficiency, electrocatalytic activity

## Abstract

In this work, MoS_2_ microspheres/PtCo-alloy nanoparticles (MoS_2_/PtCo-alloy NPs) were composited via a novel and facile process which MoS_2_ is functionalized by poly (N-vinyl-2-pyrrolidone) (PVP) and self-assembled with PtCo-alloy NPs. This new composite shows excellent electrocatalytic activity and great potential for dye-sensitized solar cells (DSSCs) as a counter electrode (CE) material. Benefiting from heterostructure and synergistic effects, the MoS_2_/PtCo-alloy NPs exhibit high electrocatalytic activity, low charge-transfer resistance and stability in the cyclic voltammetry (CV) and electrochemical impedance spectroscopy (EIS) test. Meanwhile, a high power-conversion efficiency (PCE) of 8.46% is achieved in DSSCs with MoS_2_/PtCo-alloy NP CEs, which are comparable to traditional Pt CEs (8.45%). This novel composite provides a new high-performance, stable and cheap choice for CEs in DSSCs.

## 1. Introduction

DSSCs have been regarded as one of possible alternatives to silicon photovoltaics, owing to their sustainable energy and competent power-conversion efficiency (PCE), low cost, facile fabrication process and environmental friendliness [1,2]. Normally, a sandwich structure of DSSCs consists of a photoanode sensitized by dye sensitizers, counter electrodes and electrolytes containing triiodide/iodide (I_3_^−^/I^−^). There have been extensive studies of the essential components of DSSCs, such as photoanodes [3,4], dye sensitizers [5,6,7], electrolytes [8,9] and counter electrodes (CEs) [10,11]. Among them—as the catalyzers of redox couple and electrodes—CEs are expected to have imperative characters, such as high electrocatalytic activity, good electron-conduction ability and stability [12,13]. Platinum (Pt) with excellent performance on conductivity and electrocatalytic activity has been widely used as CE materials [14]. However, the traditional iodine-based electrolyte can corrode Pt into PtI_4_ on the surface of electrode [15,16] leading to relatively low PCE and instability of DSSCs. Moreover, the high price and high temperature annealing of Pt CEs limits the industrialization of DSSCs. To alternate Pt CEs, many kinds of materials have been developed, such as cheap metals [17], alloys [18,19,20,21,22,23,24,25,26], polymers [27,28], carbon materials [29,30,31,32] and transition metal compounds [33,34,35].

Developing composite counter electrodes which consist of two or more materials are novel and effective way to improve the performance and lower the cost [36]. Generally, composite CEs can be divided into Pt-loaded composite CEs and Pt-free composite CEs. Normally, Pt-loaded composites are made by loading Pt nanoparticles on the low-cost materials. In this way, the electrocatalytic activity of composites are improved. Pt-free composites are the combination of carbon materials, transition metal compounds and polymers. Generally, the electrocatalytic activity of Pt-free composites is higher than single materials due to synergetic effects of diverse materials in composites.

Molybdenum sulfide (MoS_2_) as a typical transition metal compound possesses many attractive properties, such as a triatomic layer structure, atomic-scale thickness, high catalytic activity and outstanding stability [36,37]. MoS_2_ is mainly applied in the field of hydrodesulfurization (HDS) [38] and hydrogen evolution reaction (HER) [39] as a high-performance catalyst because there are active sites for catalyst on the edges of triatomic layers [40]. For DSSCs, Wu et al. synthesized leaf-like MoS_2_ as CEs which exhibited prominent catalytic activity for I_3_^−^/I^−^. In this research, the DSSCs with MoS_2_ CEs showed the PCE of 7.59%, comparable to that of Pt (7.64%) [33]. Recently, in addition, many further studies concentrated on MoS_2_ composited with inorganic compounds or carbonaceous materials, because the edges of MoS_2_ layers restrict the number of active sites. Through hybridizing or compositing MoS_2_ with other materials, such MoS_2_/C [41], MoS_2_/graphene [42,43], MoS_2_/CNTs [44], MoS_2_/NiS [45], Co–Mo–S [46], Fe–Co-MoS_2_ [47] could achieve higher catalytic activity and lower charge resistance.

On the other side, thanks to the outstanding electrocatalytic activity and relatively low cost of nano alloys (NAs), many kinds of NAs have been used to substitute for traditional Pt CEs [48]. NAs which are metallic materials synthesized by several metals or metals/metalloids on the nanoscale are pretty different from single metals and bulk metals on characters and catalysis performance. For NAs, many properties are better than single metals and bulk metals on the field of electrocatalysis, such as high surface energy, plentiful active sites and stability. These properties are associated with nanosized effects, abundant defects and reconfigurable electronic structures, respectively [49]. As for the application of NAs in the CEs of DSSCs, numerous kinds of NAs not only displayed excellent catalytic activity, chemical stability and synergistic effects, but also effectively reduced the costs of CEs. NAs include Pt-transition metal alloys: PtRu [50], PtCo [18], PtNi [51,52], PtFe [53,54], PtMo [55,56], PtCr [55], PtPd [55,57], PtZn [58], PtCoFe [59], PtCoNi [19]; Pt-free alloy: CoSe [60], CoNi [61], NiSe [62], CoPd [63], FeNi [64], FeSn [65].

To date, both NAs and MoS_2_ exhibits high performance as the CEs of DSSCs, however, few studies have investigated the MoS_2_/PtCo-alloy nanoparticles (NPs) composite CEs. In this article, MoS_2_ microspheres with the flake-like surface were facilely synthesized by the hydrothermal route. PtCo-alloy NPs were prepared via the co-reduction. Moreover, the uniform and controllable self-assembly between MoS_2_ and PtCo-alloy NPs based on hydrogen bonding networks was achieved by poly (N-vinyl-2-pyrrolidone) (PVP). The novel composite shows more active sites, excellent electrocatalytic activity and stability through electron microscopy, electrochemical impedance spectroscopy (EIS) and cyclic voltammetry (CV). A notable PCE of 8.46% was achieved and it is comparable to that of the DSSCs with Pt CEs. The synthesis strategy of the MoS_2_/PtCo-alloy NP composite is effective and also have promising prospects in area such as electromagnetic shielding [66] and solar to steam generation [67,68,69].

## 2. Materials and Methods

### 2.1. Materials and Reagents

Thiourea (CH_4_N_2_S), sodium molybdate (Na_2_MoO_4_·2H_2_O), chloroplatinic acid hexahydrate (H_2_PtCl_6_·6H_2_O), cobalt(II) chloride (CoCl_2_), sodium borohydride (NaBH_4_), ethylene glycol (C_2_H_6_O_2_), titanium(IV) tetrachloride (TiCl_4_), acetonitrile (C_2_H_3_ N) and lithium perchlorate (LiClO_4_) were purchased from Shanghai Aladdin Bio-Chem Technology Co., Ltd. (Shanghai, China). Poly (N-vinyl-2-pyrrolidone) (PVP K30) was purchased from Beijing Solarbio Biologic Technology Co., Ltd. (Beijing, China) 4-tert-butyl-pyridine (TBP), 1,3-dimethylimidazolium (DMII), iodine (I_2_), guanidinium thiocyanate (GNCS) and lithium iodide (LiI) were purchased from Sigma-Aldrich (St. Louis, MO, USA). All agents are analytical grade and used without any purification. The sensitized dye N719 was purchased from Dyesol. FTO glass (2.2 mm, 15 Ω/square, Nippon Sheet Glass, Tokyo, Japan) was divided into rectangles with a size of 2 × 1.5 cm^2^ and successively washed in detergent, deionized water and ethanol with the ultrasonic bath.

### 2.2. Synthesis of MoS_2_ Microspheres

The method of synthesizing MoS_2_ microspheres by using micelle template is described previously [50]. First, the PVP solution was obtained by dissolving 0.4 g of PVP (K30) into 80 mL of deionized water and stirring 30 min. Then, 1.92 g of thiourea and 0.96 g of Na_2_MoO_4_·2H_2_O were dissolved into as-prepared PVP solution by stirring 30 min. Next, the reaction solution was transferred into a 100-mL Teflon-lined stainless-steel autoclave and heated for 24 h at 200 °C. After the hydrothermal reaction finished, the black dispersion was collected by centrifugation at 7000 rpm for 15 min, wished two times with deionized water and dried at 60 °C in vacuum.

### 2.3. Synthesis of the MoS_2_/PtCo-Alloy NP Composite Material

The detailed strategy of fabricating the MoS_2_/PtCo-alloy NP composite is as follows 0.5 g of as-prepared MoS_2_ was dispersed into ethylene glycol (EG) by stirring 30 min. Then, 0.1 g of PVP was dissolved into the dispersion. After stirring 30 min, a certain amount of H_2_PtCl_6_ and CoCl_2_ (molar ratio of H_2_PtCl_6_:CoCl_2_ = 1:1) was added into the mixture with stirring and ultrasonic bath for 30 min. Then, a full amount of NaBH_4_ was gradually added into the prepared mixture. After stirring for 5 h, the composite was extracted by centrifuging and washing the mixture. The obtained composite was finally dried at 60 °C for 12 h in vacuum.

### 2.4. Preparedness of Counter Electrodes

The fabrication of MoS_2_-based CEs and Pt CEs follows different methods. The preparation of MoS_2_-based CEs was adhered to the precedent research [25]. The as-prepared MoS_2_-based materials was mixed with carbon black and PVDF at the ratio of 8:1:1 (mass ratio). The mixture was dispersed into NMP. Moreover, the dispersion was grinded until the dispersion turned to slurry with appropriate viscosity. Then, the doctor blade method was used to coat the as-obtained slurry on FTO glass. In this method, prepared solution was dropped on FTO glass, then the doctor blade was used to scrape dropped solution to form smooth and uniform film on the surface of FTO. Finally, the obtained glass with solution film heated at 500 °C for 10 min with a heat gun, in this way, CE was fabricated successfully. The material-coated FTO counter electrodes were dried at 60 °C for 12 h in vacuum and were annealed at 500 °C for 2 h in argon.

Pt CEs as a matched group in our experiments were fabricated as a traditional method. Brief, 10-mM H_2_PtCl_6_ in isopropanol was coated on FTO glass by doctor blade method and heated at 500 °C for 10 min with a heat gun.

### 2.5. Fabrication of DSSCs

The process of photoanodes and DSSCs fabrication was described like previously studies [70,71]. To begin with, a TiO_2_ blocking layer was formed on FTO glass by soaking washed FTO glass into a 40-mM TiCl_4_ aqueous solution and heating at 70 °C for 30 min. Afterwards, a TiO_2_ layer was coated on the pretreated FTO glass by screen printing. The thickness of TiO_2_ layer was controlled by the times of repeating the screen printing. Subsequently, the TiO_2_ film was roasted at 450 °C for 30 min and natural cooled in air. When it cooled down to 120 °C, the anode was immersed into 0.5-mM N719 acetonitrile and tert-butanol (1/1, *v/v*) solution for 24 h. Next, as-prepared anodes and counter electrodes were attached to the sandwich structure by hot-pressing with Surlyn films. Then, the iodine-based electrolyte which is the acetonitrile solution composed of 0.60-mM DMII, 0.03-M I2, 0.10-M GNCS and 0.50-M TBP was injected into the sandwich structure.

### 2.6. Characterization

The micrographs of materials were taken by the field-emission scanning electron microscope (FESEM, HITACH SU70, Tokyo, JPN) and the high-resolution transmission electron microscope (HRTEM, FEI, Tecnai G2 F30). X-ray diffraction (XRD) analysis was achieved by an X-ray diffractometer (Empyrean, Panalytical, Malvern, UK) with Cu-Kα radiation. A X-ray photoelectron spectrometer (ESCALAB250Xi, Thermo Fisher, Waltham, MA, USA) was employed in finishing the X-ray photoelectron spectra (XPS) analysis.

### 2.7. Electrochemical and Photovoltaic Measurements

All the electrochemistry measurements were implemented on an electrochemistry workstation (CHI660e, Chenhua instruments Ins., Shanghai, China). The cyclic voltammetry (CV) was conducted to estimate the performance of counter electrodes. This measurement system consists of three electrodes (a Pt auxiliary electrode, an Ag/AgCl reference electrode and a 3 cm^2^ of working electrode) and iodine-based electrolyte which is the acetonitrile solution containing 10-mM LiI, 1-mM I_2_ and 0.1-M LiClO_4_. The electrochemistry impedance spectroscopy (EIS) was measured with symmetric dummy cells composed of double identical counter electrodes. Moreover, they were injected the electrolyte which was same as the DSSCs electrolyte in our experiments. The active area of dummy cells was controlled at 0.64 cm^2^.The frequency range of EIS was from 1 Hz to 10^5^ Hz. Moreover, tests were at 0.01 V of bias voltage and in dark. The photocurrent density–voltage (*J–V*) curves of the DSSCs were measured by the electrochemistry workstation. The simulative solar light (100 mW·cm^−2^, AM 1.5) was produced by a Xe lamp (cel-S500/350, Beijing China Education Au-light Co., Ltd., Beijing, China).

## 3. Results

**Figure 1:**
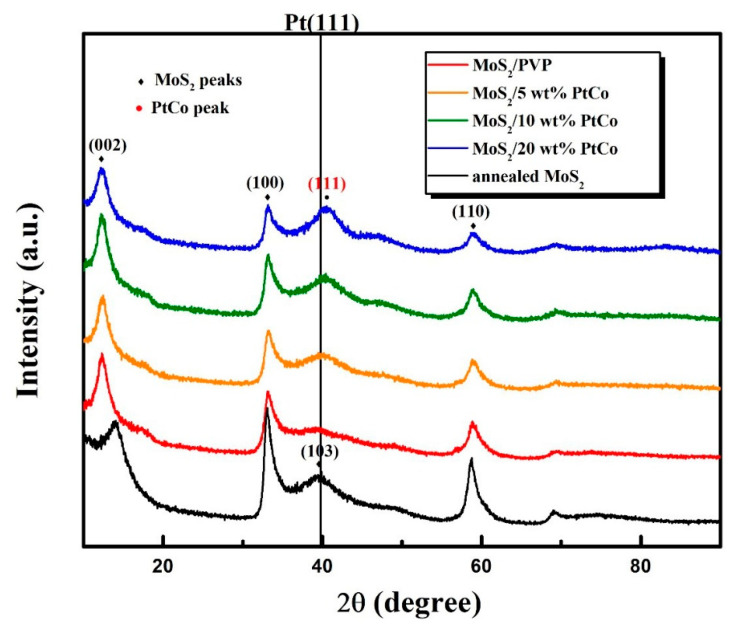
XRD patterns of MoS_2_ microspheres and MoS_2_/PtCo-alloy nanoparticle (NPs) composites.

To investigate the crystal structure of samples, a series of XRD tests were conducted. The XRD patterns of MoS_2_ microspheres and MoS_2_/PtCo-alloy NP composites are shown in Figure 1.

Among different types of samples, MoS_2_/PVP means the fresh material which was synthesized by the PVP micelle-assisted hydrothermal route and covered by molecular chains of PVP. Moreover, annealed MoS_2_ was heated at 500 °C for 2 h in argon to remove PVP which can influence the shape and peak positions of XRD patterns. In particular, featured peaks of MoS_2_ at 14.02° (002 planes) reflect the condition of MoS_2_ layer stacking (strong peak indicates order stacking of MoS_2_ layer) [72]. In Figure 1 the (002) peaks of fresh samples shift towards lower angles and that peak of the annealed sample returns to 14.02° and is diminished. The reason of peak shift is molecular chains of PVP between MoS_2_ layers increase the average spacing between layers. Moreover, the peak of annealed sample is weakened, because residual carbon produced by burning PVP turned closely the stacking structure of MoS_2_ to the approximately single-layer structure. Meanwhile, the MoS_2_/PtCo-alloy NP composites which contain different amount of PtCo-alloy NPs are denoted as MoS_2_/5 wt% PtCo, MoS_2_/10 wt% PtCo and MoS_2_/20 wt% PtCo. The peaks of XRD patterns roughly accord with figures of hexagonal MoS_2_ peaks (JCPDS card No.37–1492) and the peak shifts occur obviously on (002), (100) and (110) MoS_2_ planes. Peak position shifts apparently associate with macromolecular chain of PVP adhering, because the peak positions of MoS_2_ become more accordant for the standard hexagonal MoS_2_ pattern after samples were removed PVP by annealing. The (111) diffraction peak of PtCo-alloy NPs is also exhibited in Figure 1. A distinct shift of the Pt (111) diffraction peak towards a higher degree owing to the shrinking lattices of PtCo. Pt atoms substituted by smaller Co atoms normally leads to contraction of PtCo lattices which coincides with previous results [73].

**Figure 2:**
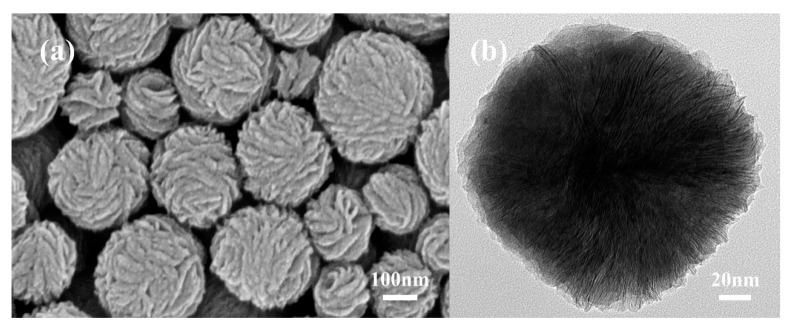
Morphologic characterizations of MoS_2_ microspheres prepared by the poly (N-vinyl-2-pyrrolidone) (PVP) micelle-assisted hydrothermal method. (**a**) FESEM image; (**b**) low-resolution TEM image.

**Figure 3:**
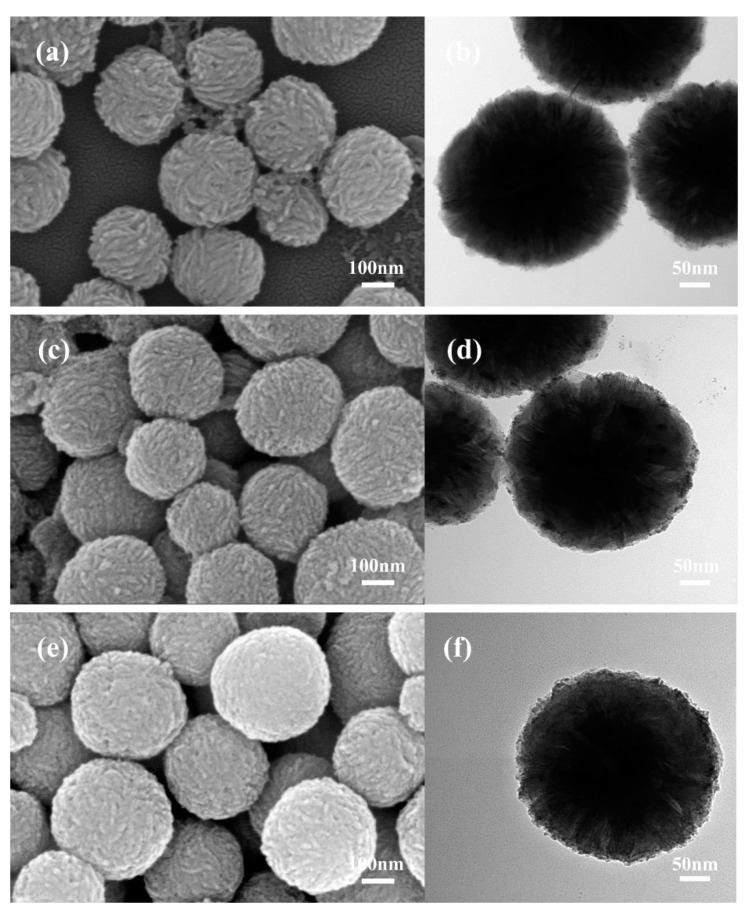
FESEM and TEM images of MoS_2_/PtCo-alloy NP composites (20 wt% PtCo-alloy NPs loading) prepared by different methods. (**a**,**b**) Co-reducing PtCo alloy directly; (**c**,**d**) adding PVP before co-reduction in aqueous; (**e**,**f**) adding PVP before co-reduction in ethylene glycol.

FESEM and TEM images of MoS_2_ microspheres and MoS_2_/PtCo-alloy NP composites synthesized by different methods are shown in Figure 2 and Figure 3, respectively. MoS_2_ microspheres which are composed by vertical MoS_2_ nanoflakes expose edges of nanoflakes on the surface of microspheres. This structure is beneficial to improve the catalytic activity because more edges are exposed on the surface of MoS_2_ microspheres. In the TEM images of MoS_2_ microspheres, many molecular chains of PVP covered microspheres. The PVP-functionalized MoS_2_ microspheres create the precondition for self-assembly of MoS_2_/PtCo-alloy NP composites.

To synthesize the MoS_2_/PtCo-alloy NP composites, three different routes were conducted in our experiments, including co-reducing PtCo-alloy NPs directly, adding PVP before co-reduction in aqueous and adding PVP before co-reduction in ethylene glycol (Figure 3). Co-reducing PtCo-alloy NPs in the MoS_2_ and PtCo precursors directly (Figure 3a,b) leads to less alloys loaded and obvious aggregation. To increase the amount of loaded alloys and reduce nanoparticles aggregation, we added PVP into the mixed dispersion before co-reduction (Figure 3c,d). PVP can keep noble metal nanoparticles from aggregation [74]. Moreover, functionalized PtCo-alloy NPs can trend to self-assemble on the surface of MoS_2_, because the hydrogen-bond networks which is established by hydrophilic groups of PVP can draw PtCo-alloy NPs on the surface of MoS_2_ microspheres. Moreover, ethylene glycol as the solution of reduction reaction can improve reducing effectiveness and dispersibility [75]. Thus, more uniform, highly dispersed and vast PtCo-alloy NPs loaded on MoS_2_ microspheres (Figure 3e,f).

**Figure 4:**
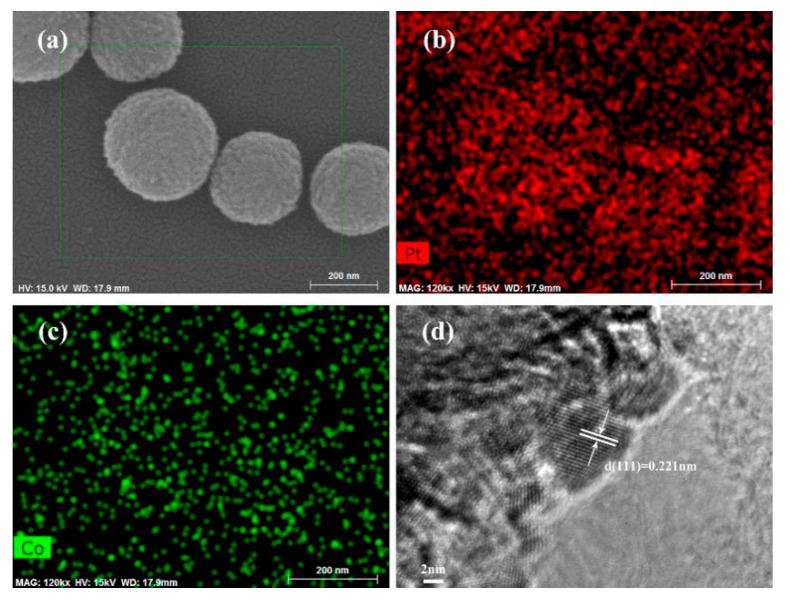
EDS mapping images of the MoS_2_/PtCo-alloy NP composites. (**a**) Mapping area of SEM image; (**b**) element mapping of Pt; (**c**) element mapping of Co; (**d**) HRTEM image of PtCo-alloy NPs.

**Figure 5:**
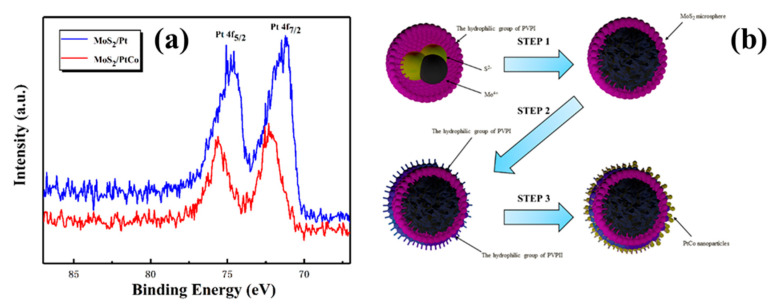
(**a**) Pt 4f XPS spectra of MoS_2_/PtCo-alloy NP composites and MoS_2_/Pt NPs composites; (**b**) schematic diagram of the process of synthesizing MoS_2_/PtCo-alloy NP composites.

EDX mapping images of MoS_2_/PtCo-alloy NPs depict that Pt and Co were uniformly dispersed on the surface of MoS_2_ microspheres as shown in Figure 4b,c, respectively. Additionally, the HRTEM image and XPS further demonstrate the formation of PtCo-alloy NPs. The HRTEM (Figure 4d) indicates the (111) interplanar distance of 0.221 nm was smaller than 0.226 nm which is interplanar spacing of standard Pt (111). This is the other proof that Co atoms enter into the lattices of Pt [76]. Moreover, the Pt 4f peak shifted to higher energies can be obviously observed on the XPS in Figure 5a. This phenomenon is always associated with alloy formation with Co [77]. There were different work function in Pt and Co. When the combination of them were made to form PtCo alloy, higher atomic ratio of Co to Pt lead to the rehybridization of the d-band and the sp-band, hence, as the reference level in XPS measurements, the change in Fermi level result in the shifting to higher binding energy of Pt 4f peak [78].

A typical synthesis route of MoS_2_/PtCo-alloy NP composites is shown in Figure 5b. Three steps can be concluded for the synthesis of MoS_2_/PtCo-alloy NP composites. First, micelle-assisted hydrothermal method was carried out to synthesize MoS_2_ microspheres covered by PVP. This special MoS_2_/PVP hybrids are easy to disperse and draw more molecular chains of PVP to form the hydrogen bonded networks, because hydrophilic amide groups of PVP on the surface of MoS_2_ point outward [62]. Thus, when PVP is added for the second time, the new PVP is attracted onto hydrophilic amide groups of primary PVP. Moreover, the new PVP stabilizes PtCo-alloy NPs through coordinating Pt atoms with the N and O atoms of PVP simultaneously [79]. When the co-reduction finishes, PtCo-alloy NPs disperse around MoS_2_ microspheres. The new PVP acts as “a bridge” to self-assemble PtCo-alloy NPs on the surface of MoS_2_.

During the process of preparing CEs with doctor blade method, we found that with 5 wt% PtCo and 12 wt% PtCo, surface tension of liquid is large and not easy to spread, so it cannot be wetted and converged on conductive glass. According to repeated experiments, the slurry with 20 wt% PtCo is the easiest to be coated uniformly on the electrode and its performance is also the most ideal. Therefore, CE with 20 wt% PtCo is used for the subsequent electrochemical catalytic activity and photovoltaic performance.

### 3.1. Cyclic Voltammetry Analysis

**Figure 6:**
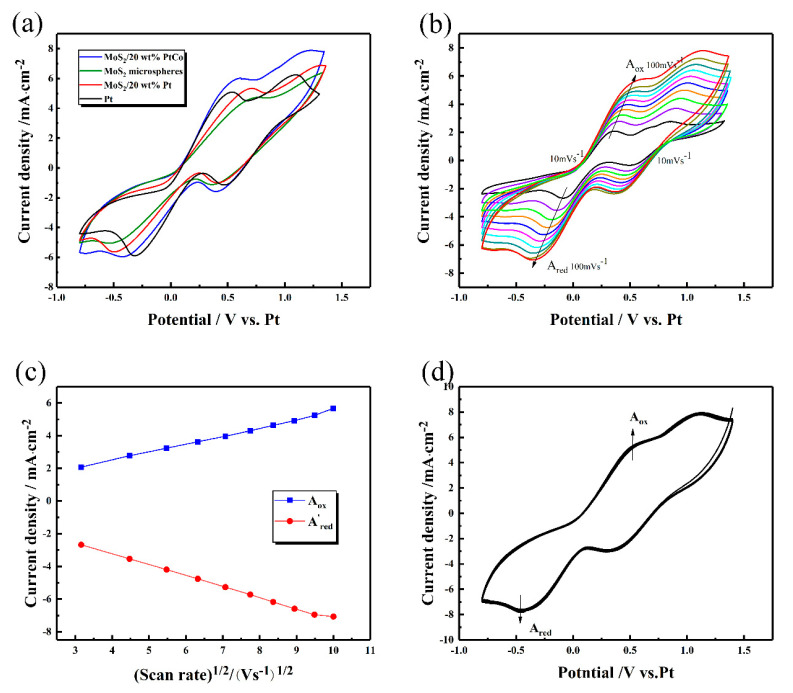
(**a**) Cyclic voltammetry (CV) for counter electrodes based on the different materials; (**b**) CVs for the MoS_2_/PtCo-alloy NP composites with different scan rates (from 10 mV s^−1^ to 100 mV s^−1^, at the intervals of 10 mVs^−1^); (**c**) relationship of redox peak current density and square root of scan rate; (**d**) 100-times continuous cycle scan CVs for the MoS_2_/PtCo-alloy NP composite CEs at a scan rate of 100 mV s^−1^.

Due to the notable improvement of PtCo loading in cells performance, the composites with maximum PtCo loading (MoS_2_/20 wt% PtCo) in this paper was chosen as the representation of MoS_2_/PtCo-alloy NP composites to achieve CV and EIS test. At same time, for better lateral comparison, the loading content of pure Pt NPs in MoS_2_/Pt NPs is also 20 wt%. Cyclic voltammetry (CV) curves (Figure 6a) of MoS_2_/PtCo-alloy NP composites, MoS_2_/Pt NPs composites, MoS_2_ microspheres, and traditional Pt electrodes in I_3_^−^/I^−^ electrolyte were measured at a scan rate of 100 mV s^−1^ from −0.8 V to 1.4 V. Two couples of oxidation and reduction peaks are detected. The high potential peak which can be ascribed to the Reaction (1). Moreover, the low potential peak which is mainly related to performance of DSSCs is attributed to the Reaction (2) [80].

2I_3_^−^ ↔ 3I_2_ + 2e^−^ (1)

3I^−^ ↔ I_3_^−^ + 2e^−^ (2)

The cathodic peaks potentials of MoS_2_/PtCo-alloy NP composites, MoS_2_/Pt NPs composites, MoS_2_ microspheres and pure Pt electrodes are −0.415 V, −0.495 V, −0.520 V and −0.320 V, respectively. The cathodic peaks potential of MoS_2_/PtCo-alloy NP CEs is slightly larger than that of Pt CEs, but the redox current density of MoS_2_/PtCo-alloy NP CEs is slightly larger than Pt CEs. These indicators show MoS_2_/PtCo-alloy NP CEs possess similar electrocatalytic activity and reaction velocity to Pt CEs.

The charge-transfer mechanism of the I_3_^−^/I^−^ system on CE was investigated by scanning CVs at series of scan rates (Figure 6b). For MoS_2_/PtCo-alloy NP composite CEs, the cathodic peaks (corresponding to I_3_^−^ + 2e^−^ ↔ 3I^−^) and the anodic peaks (corresponding to 3I_2_^−^ + 2e^−^ ↔ 2I_3_^−^) regularly shifted towards negative direction and positive direction, respectively. The linear relationship between redox peak current and square root of scan rate is shown in Figure 6c. This relationship is acquired by measuring CVs with different scan rates and record the current density of peaks. It indicates only diffusion of I_3_^−^/I^−^ ion pair limits redox reactions between the MoS_2_/PtCo-alloy NPs CE and electrolyte [81].

Taking repeated CV scans of CEs is a routine method to simulate DSSCs in a long time running. The extent of peak shifts in CVs curves strongly associated with electrocatalysis reduction and chemical degradation. The 100 times continuous CVs for MoS_2_/PtCo-alloy NP composites is shown in Figure 6d. The shape of the CVs curve is highly stable through repeated CVs test, attributing to the excellent electrochemical stability of MoS_2_/PtCo-alloy NP composites.

### 3.2. Electrochemical Impedance Analysis

**Figure 7:**
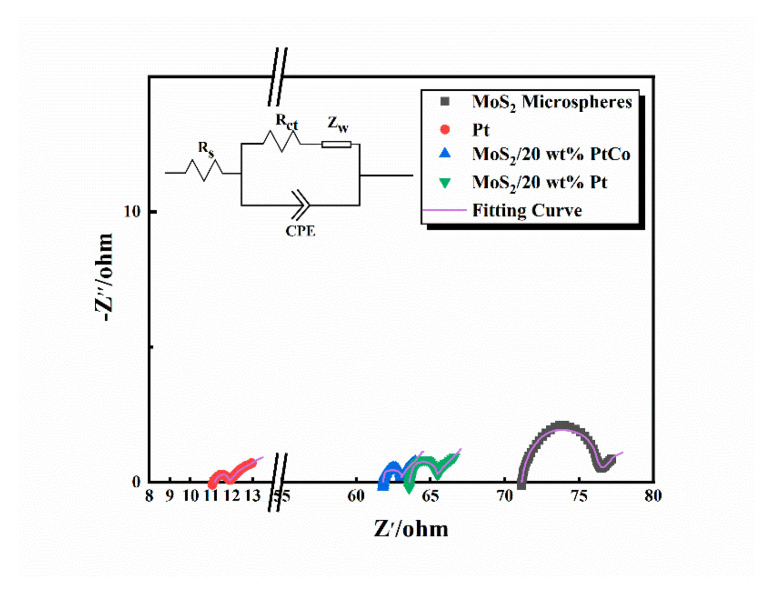
Electrochemical impedance spectroscopy (EIS) of dummy cells fabricated with two identical MoS_2_/20 wt% PtCo, MoS_2_/20 wt% Pt, MoS_2_ microspheres, conventional Pt counter electrodes (CEs) and corresponding fitting curves. The equivalent circuit model is inserted in it.

Electrochemical impedance spectra (EIS) is an important tool to analyze the dynamics of reactions in cells and the surface construct of CEs. The internal resistances of DSSCs are usually divided into series resistance (*R*_s_), charge-transfer resistance (*R*_ct_), diffusion resistance (*Z*_w_) and constant phase element (CPE). DSSC with MoS_2_-based CEs had higher *R*_s_ values, which could be ascribed to the different preparation technology. The diffusion of iodine-based electrolytes in the alloy composite is so rapid that, over time, the electrolyte diffuses out of the battery. In order to solve this problem, a circular CE smaller than the package area of the battery was prepared. As result of changing the location and area of CE, the electronic transmission of such electrodes was not just through CE being injecting into the electrode clamp, but passing from CE and FTO in turn, then flowing into the electrode clamp. The FTO was less conductive than CE, therefore, the existence of this process in MoS_2_-based CEs lead to higher *R_s_* compared with Pt CE. Among them, the *R*_ct_ of CE/electrolyte is an index to evaluate the electrocatalytic activity, because the small *R*_ct_ signifies low overpotential and high electron transferring bet ween electrolyte and CEs. Figure 7 shows the Nyquist plots of dummy cells consisted of the CE/electrolyte/CE structure. The *R*_ct_ values of MoS_2_/20 wt% PtCo is 1.04 Ω, which is proximity to that of pure Pt (0.71 Ω), indicating that the electrocatalytic activity of MoS_2_/PtCo is close to that of pure Pt. Moreover, the *R*_ct_ values of MoS_2_/20 wt% PtCo is smaller than that of MoS_2_/20 wt% Pt (1.76 Ω) and MoS_2_ microspheres (5.18 Ω). It could be put down to the improvement of catalytic ability and electron transfer ability as the result of the metal nanoparticles filling in lamellar interstitial space. Furthermore, synergistic catalysis also plays a role in reduction of *R*_ct_. The competent charge-transfer ability of MoS_2_/20 wt% PtCo CEs displays in the EIS, owing to synergistic effects and MoS_2_/PtCo-alloy NPs heterostructure.

### 3.3. Photovoltaic Performance of DSSCs

**Figure 8:**
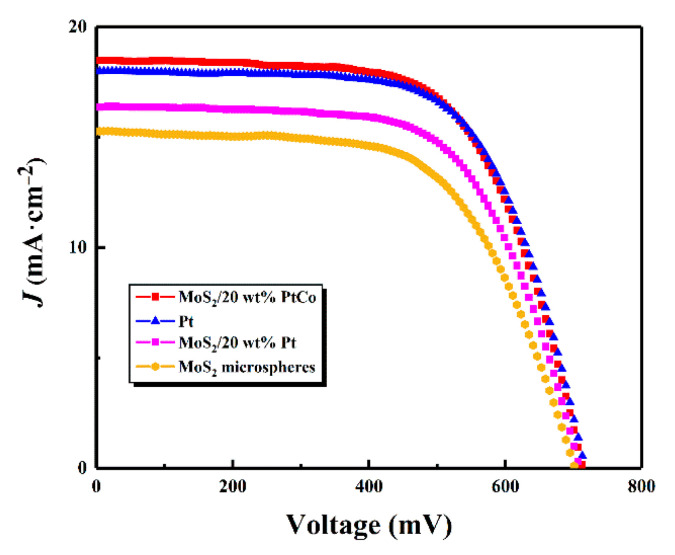
*J–V* curves of dye-sensitized solar cells (DSSCs) with different counter electrodes.

Figure 8 shows the *J*–*V* curves of DSSCs with CEs based different materials. Moreover, all the paraments of photovoltaic performance are exhibited in Table 1. When the MoS_2_/20 wt% PtCo-alloy NP composite was applicated on the DSSCs as the CE, the DSSCs achieved the impressive conversion of 8.46%, similar to that of Pt CEs. Meanwhile, the DSSCs with the MoS_2_/PtCo-alloy NP composite CEs possess high short-circuit current (*J*_sc_), open-circuit voltage (*V_oc_*) and fill factor (FF) which cannot be gained on the DSSCs with MoS_2_ CEs. It means there is a significant improvement on the electrocatalytic activity of materials by loading PtCo-alloy NPs on the surface of MoS_2_. The change in *V_oc_* could also be explained by the result of CV. According to formula, the change in redox energy level could influence open-circuit voltage with the relationship of equation:

$$V_{oc}\;=\;\frac{KT}{e}ln\frac{J_{inj}}{qek_{et}c_{ox}N_{c}}\;+\;\frac{E_{ref}}{e}$$

In which *K* was molar gas constant, *T* was temperature, *e* was elementary charge and *J* was current density of photogenerated electrons. Symbol *q* represented the number of electrons during the process of charge transfer with corresponding concentration *c_ox_* and transfer rate *k_et_*. *N_c_* was the density of states of TiO_2_ conduction band. *E_ref_* could be obtained by taking the difference between conduction band edge and the redox potential of the redox coupling electrolyte. Half-wave potentials of MoS_2_, MoS_2_/Pt, Pt and MoS_2_/PtCo CEs measured in CV were 121 mV, 110 mV, 105 mV and 92 mV, respectively, showing the negative shift trend. Therefore, the most negative shift of MoS_2_/PtCo CEs in the I_3_^–^/I^–^ redox energy level resulted in the increase of *V_oc_* in DSSCs, leading to the highest *V_oc_* [82]. The results of CV showed great consistency in terms of *J–V* curve. The performance gap between MoS_2_/PtCo CEs and MoS_2_/Pt CEs is also observed in Figure 8, because the change of electron structure improves the electrocatalytic activity and charge-transfer ability [49].

## 4. Discussion

A new and simple self-assembly method was conducted to synthesize the MoS_2_/PtCo-alloy NP composite, which was applied to DSSCs as CEs. PVP functionalizes both MoS_2_ and PtCo-alloy NPs and links each other by hydrogen bonded networks. Finally, the morphology tests investigate that uniform and mass PtCo-alloy NPs were loaded on the surface of MoS_2_. The structure and composition of MoS_2_/PtCo-alloy NP composites were studied by XRD, EDS and XPS to prove that PtCo-alloy NPs successfully load on MoS_2_. Furtherly, the result of CVs and EIS shows the low charge-transfer resistance, outstanding electrocatalytic activity and excellent stability of this material, which is owing to more PtCo-alloy NPs active sites provided and good intrinsic performance of PtCo. Finally, the *J–V* curves were measured under 100 mW cm^−2^ of simulated solar illumination. Moreover, the PCE of 8.46% obtained by the DSSCs with MoS_2_/PtCo-alloy NP CEs is similar to that of DSSCs based on Pt CEs. Overall, the MoS_2_/PtCo-alloy NP composite can be regarded as an alternation for the high-cost pure Pt CEs.

## Figures and Tables

**Table 1:** Detailed photovoltaic parameters and EIS parameters of DSSCs based on different counter electrodes.

CE	*J*_sc_ (mA cm^−2^)	*V_oc_* (mV)	FF	PCE (%)	*R*_s_ (Ω)	*R*_ct_ (Ω)
Pure Pt	17.978	718	0.655	8.45	11.17	0.71
MoS_2_/20 wt% PtCo	18.487	715	0.640	8.46	61.69	1.04
MoS_2_/20 wt% Pt	16.338	710	0.639	7.41	63.72	1.76
MoS_2_ microspheres	15.247 ^1^	703 ^2^	0.616 ^3^	6.61	71.32 ^4^	5.18 ^5^

^1^*J*_sc_—short-circuit current density; ^2^*V_oc_*—open circuit voltage; ^3^ FF—fill factor; ^4^ *R*_s_—series resistance; ^5^ *R*_ct_—charge-transfer resistance.
