# Erratum: Wang, D., et al. Self-Assembly Synthesis of the MoS_2_/PtCo Alloy Counter Electrodes for High-Efficiency and Stable Low-Cost Dye-Sensitized Solar Cells. *Nanomaterials* 2020, *10*, 1725

**DOI:** 10.3390/nano10112217

**Published:** 2020-11-06

**Authors:** Zhi Zeng, Dongbo Wang, Jinzhong Wang, Shujie Jiao, Yuewu Huang, Sixiang Zhao, Bingke Zhang, Mengyu Ma, Shiyong Gao, Xingguo Feng, Liancheng Zhao

**Affiliations:** 1 Department of Optoelectronic Information Science, School of Materials Science and Engineering, Harbin Institute of Technology, Harbin 150001, China; zengzhi_1997@163.com (Z.Z.); huangyuewu703@163.com (Y.H.); auspice123@163.com (S.Z.); zhangbingke007@163.com (B.Z.); wangdong165@sina.com (M.M.); gaoshiyong@hit.edu.cn (S.G.); lczhao@hit.edu.cn (L.Z.); 2 National Key Laboratory of Science and Technology on Surface Engineering, Lanzhou Institute of Physics, Lanzhou 730000, China

## Incorrect Funding Number

There is an error in the Funding statement of [1]. The correct number for **the National Key Research and Development Program of China** is **2019YFA0705201**. The authors apologize for any inconvenience caused and state that the scientific conclusions are unaffected. The original article has been updated.
